# Sirt1 Is Required for Resveratrol-Mediated Chemopreventive Effects in Colorectal Cancer Cells

**DOI:** 10.3390/nu8030145

**Published:** 2016-03-05

**Authors:** Constanze Buhrmann, Parviz Shayan, Bastian Popper, Ajay Goel, Mehdi Shakibaei

**Affiliations:** 1 Institute of Anatomy, Ludwig-Maximilian-University Munich, Pettenkoferstrasse 11, Munich D-80336, Germany; constanze.buhrmann@med.uni-muenchen.de; 2 Department of Parasitology, Faculty of Veterinary Medicine, University of Tehran, Tehran 141556453, Iran; pshayan@ut.ac.ir; 3 Investigating Institute of Molecular Biological System Transfer, Tehran 1417863171, Iran; 4 Department of Anatomy and Cell Biology, Biomedical Center, Ludwig-Maximilian-University Munich, Martinsried D-82152, Germany; Bastian.Popper@med.uni-muenchen.de; 5 Center for Gastrointestinal Research; Center for Epigenetics, Cancer Prevention and Cancer Genomics, Baylor Research Institute and Sammons Cancer Center, Baylor University Medical Center, Dallas, TX 75246, USA; AjayG@BaylorHealth.edu

**Keywords:** human colon cancer, Sirt1, NF-κB, alginate, resveratrol, carcinogenesis

## Abstract

Sirt1 is a NAD^+^-dependent protein-modifying enzyme involved in regulating gene expression, DNA damage repair, metabolism and survival, as well as acts as an important subcellular target of resveratrol. The complex mechanisms underlying Sirt1 signaling during carcinogenesis remain controversial, as it can serve both as a tumor promoter and suppressor. Whether resveratrol-mediated chemopreventive effects are mediated via Sirt1 in CRC growth and metastasis remains unclear; which was the subject of this study. We found that resveratrol suppressed proliferation and invasion of two different human CRC cells in a dose-dependent manner, and interestingly, this was accompanied with a significant decrease in Ki-67 expression. By transient transfection of CRC cells with Sirt1-ASO, we demonstrated that the anti-tumor effects of resveratrol on cells was abolished, suggesting the essential role of this enzyme in the resveratrol signaling pathway. Moreover, resveratrol downregulated nuclear localization of NF-κB, NF-κB phosphorylation and its acetylation, causing attenuation of NF-κB-regulated gene products (MMP-9, CXCR4) involved in tumor-invasion and metastasis. Finally, Sirt1 was found to interact directly with NF-κB, and resveratrol did not suppress Sirt1-ASO-induced NF-κB phosphorylation, acetylation and NF-κB-regulated gene products. Overall, our results demonstrate that resveratrol can suppress tumorigenesis, at least in part by targeting Sirt1 and suppression of NF-κB activation.

## 1. Introduction

Colorectal cancer (CRC) is one of the major leading causes of tumor-associated deaths in the world, with high rates of incidence and disease-related mortality and morbidity [1]. The pathogenesis and development of CRC is a multi-step process that is orchestrated through complex molecular signaling mechanisms, including the mutations in multiple genes, such as proto-oncogenes and tumor suppressor genes [2].

There is a growing recognition that the tumor microenvironment is very complex and contains different cell types and factors, which play a critical role during the initiation, proliferation, metastasis and maintenance of cancers [3,4,5,6]. Moreover, several lines of evidence have shown that the transcription factor, nuclear factor-kappa B (NF-κB), is a critical determinant, as it regulates a variety of pathophysiological processes, such as inflammation, cell survival, proliferation, invasion, apoptosis, differentiation and chemoresistance in different tumor cells, including CRC [7,8]. Therefore, pharmacologically-safe anti-tumor agents with the potential to influence the tumor microenvironment and inhibit NF-κB signaling activation may enhance chemosensitivity and reduce metastasis of tumor cells and provide a promising approach for the prevention or treatment of tumors.

Resveratrol (3,5,4′-trihydroxystilbene), a naturally-occurring polyphenolic and multi-targeted component that belongs to the class of stilbenes present in more than 70 plant species, including skin of red grapes, berries and peanuts [9,10,11], has been shown to act as a specific inhibitor of NF-κB in different tumor cells [12,13]. Several lines of evidence have shown the anti-oxidant, anti-inflammatory and neuroprotective properties of resveratrol [14], leading to its cancer chemopreventive and chemotherapeutic activity in various cancer cell lines [9]. Several reports have shown that resveratrol has inhibitory effects on various types of cancer cell lines and preclinical animal models of various cancers, such as colon, breast and lymphoma, through its activity on diverse subcellular targets, including Sirt1 [15,16,17,18,19,20,21]. Sirtuins (SIRT1 to SIRT7), the class III histone/protein deacetylases (HDACs), have been shown to regulate a variety of cellular processes involved in metabolism, differentiation, inflammation, aging, apoptosis, proliferation and cell cycle regulation. The best identified and characterized among the human sirtuins is sirtuin1 (silent information regulator-1, SIRT1), a nuclear enzyme, that has been shown to regulate important metabolic and physiological processes that play a critical role in processes related to cancer [22,23,24,25,26]. Furthermore, it has been reported that acetylation and deacetylation of proteins is a hallmark of the epigenetic gene expression modulation in tumorigenesis [27,28,29,30]. This enzymatic protein modulation is also post-translationally very important for the regulation of the function of many different proteins [31,32]. Resveratrol is a specific and potent activator of subcellular target histone deacetylase Sirt1 in different human tissues, including human tumors [17,33,34,35,36].

There is still poor understanding of whether Sirt1 acts as a tumor promoter through induction of genomic instability in cancers [37,38,39,40,41], possibly through deacetylation and suppression of p53 activity [42,43], and/or modulating the activity of various tumor suppressing proteins in different cancer types, including colon cancer. Furthermore, Sirt1 may act as a tumor suppressor through improving genetic stability, as shown in *in vivo* normal tissue cells, and in addition to that, Sirt1 regulates other signaling mechanisms. Indeed, it has been reported that Sirt1 blocks NF-κB signaling pathway activation, which induces inflammation and tumor invasion [42,43,44,45,46,47,48]. Furthermore, the hallmarks of tumor are the genetic instability of tumor cells, whereas healthy cells with intact innate signaling pathways are able to antagonize cancer-promoting signals and are able to resolve any cancer-promoting signals [49]. Apparently some genes, including sirtuins, may function in a context-dependent manner, including conditions, such as tumor microenvironment, divergent cellular p53 status and origin of the tumor, to exert tumor-promoting or -suppressing qualities [49].

We hypothesize that transcriptional modulation of Sirt1 regulates one of the key mechanisms of the resveratrol-mediated anti-tumorigenic effects in CRC cells. To examine this hypothesis, we evaluated an *in vitro* 3D-model culture of carcinogenesis to study the effects of resveratrol targeting Sirt1 with specific antisense oligonucleotides (ASO) on cellular proliferation, invasion and NF-κB signaling pathways in human CRC cells.

## 2. Experimental Section

### 2.1. Antibodies

Polyclonal antibodies against Sirt1 and CXCR4 were purchased from Abcam PLC (Cambridge, UK). Anti-phospho-specific p65 (NF-κB) and anti-phospho-specific p50 (NFκB) were obtained from Cell Technology (Beverly, MA, USA). Anti-MMP-9 was purchased from R&D Systems, Inc., (Heidelberg, Germany). Anti-Ki-67 and secondary antibodies used for fluorescence labelling were purchased from Dianova (Hamburg, Germany). Monoclonal poly(ADP-ribose) polymerase (PARP) antibodies were purchased from Becton Dickinson (Heidelberg, Germany). Acetylated lysine (Ac-K-103) antibody was purchased from Cell Signaling Technology (Danvers, MA, USA). Antibodies against β-actin and Ki-67 were obtained from Santa Cruz Biotechnology (Santa Cruz, CA, USA). Alkaline phosphatase-linked sheep anti-mouse and sheep anti-rabbit secondary antibodies for immunoblotting were purchased from EMD Millipore (Schwalbach, Germany).

### 2.2. Growth Media, Chemicals and Cytokines

Cell culture growth medium consisting of Dulbecco’s Modified Eagle’s Medium/Ham’s F-12 (1:1), 10% fetal bovine serum (FBS), 0.5% amphotericin B solution, 1% penicillin streptomycin solution (10,000 IU/10,000 IU), 75 μg/mL ascorbic acid, 1% essential amino acids and 1% glutamine was obtained from Seromed (Munich, Germany). Epon was obtained from Plano (Marburg, Germany). Alginate was purchased from Sigma (Munich, Germany). Resveratrol with purity greater than 98% was purchased from Sigma. A 100 mM stock solution of resveratrol (molecular weight 228.2) was prepared in ethanol and further diluted in cell culture medium to prepare working concentrations. The maximum final content of ethanol in cultures was less than 0.1%. This concentration was also used as a control.

### 2.3. Cell Lines and Cell Culture

Human HCT116 CRC cells were obtained from the European Collection of Cell Cultures (Salisbury, UK). SW480 CRC cells were purchased from the American Type Culture Collection (ATCC, Manassas, VA, USA). The cells were maintained in tissue culture flasks in growth medium and in a humidified incubator at 37 °C in an atmosphere of 95% air and 5% CO_2_. The medium was changed every three days, and cells were passaged using trypsin/EDTA.

### 2.4. Alginate Culture and Experimental Design

A detailed description of the cell cultivation in alginate is given by Shakibaei and de Souza [50,51,52]. Briefly, the pellet of HCT116 and SW480 cells (1 × 10^6^/mL) was resuspended in sterile alginate medium (2% in 0.15 M NaCl, stirring for 1–2 h) and slowly added dropwise into a solution containing 100 mM CaCl_2_ at ambient temperature (AT). The alginate beads polymerized in the presence of CaCl_2_ after 10 min. Subsequently, the CaCl_2_ solution was removed and the alginate beads washed three times with 0.15 M NaCl solution and twice with serum-starved medium (3% FBS) before starting treatment.

### 2.5. Antisense and Lipofectin-Mediated Transfection

Transient transfection of HCT116 and SW480 cells in alginate beads was performed as described previously [53]. Phosphorothioated antisense oligonucleotide derived from the mRNA nucleotide sequence of sirtuin-1 gene (Sirt1-ASO; sequence 5′-GTATTCCACATGAAACAGACA-3′) and control sense oligonucleotides (Sirt1-SO; sequence 5′-TGTCTGTTTCATGTGGAATAC-3′) used in the experiments were synthesized by Eurofins (MWG/Operon, Ebersberg, Germany). Sirt1-ASO and Sirt1-SO were phosphorothioate-modified to protect them from the cell nucleases. Alginate beads of HCT116 and SW480 cells (1 × 10^6^/mL) were either left untreated or treated with 5 µM resveratrol, or transfected by incubation with 0.5 µM Sirt1-ASO or Sirt1-SO and 10 µL/mL Lipofectin transfection reagent (Invitrogen), or treated with various concentrations of resveratrol (1, 5, 10 µM) and co-treated with 0.5 µM Sirt1-ASO, or Sirt1-SO and 10 µL/mL Lipofectin transfection reagent in serum-starved medium (3% FCS) for 14 days. These experiments were performed in triplicate, and the results are provided as mean values from three independent experiments.

### 2.6. Cell Proliferation and Viability Assay

HCT116 and SW480 cell lines (1 × 10^6^/mL) were cultured in alginate beads in Petri dishes, treated with or without different concentrations of individual resveratrol (1, 5, 10, 20 and 50 µM) to get the IC_50_ value drugs as described previously from our group [52]. In a separate set of experiment, CRC cells were treated with different concentrations of resveratrol (1, 5, 10 µM) or co-treated with fixed (0.5 µM) Sirt1-ASO or Sirt1-SO, 10 µL/mL Lipofectin transfection reagent and with different concentrations of resveratrol (1, 5, 10 µM) in serum-starved medium for 21 days. To examine the cell viability and proliferation of colorectal cancer cells in alginate bead culture, cells were retrieved from alginate and measured using the MTT assay (3-(4,5-dimethylthiazol-2-yl)-2,5-diphenyltetrazolium bromide). To release the CRC cells from the alginate, alginate beads were washed two times with PBS and dissolved in 55 mM sodium citrate solution. To remove excess alginate, cells were centrifuged, washed twice with sterile PBS and resuspended in 2 mL modified cell culture medium (DMEM without phenol red, without ascorbic acid and only 3% FBS). Subsequently, 100 µL of cell suspension was distributed to a 96-well-plate; to each well were immediately added 10 µL MTT solution (5 mg/mL), and the plate was incubated for 4 h at 37 °C. Finally, 100 µL of the MTT solubilization solution (10% Triton x-100/acidic isopropanol) was added per well and the cells incubated overnight at 37 °C. Absorbance was measured at 550 nm (OD550) using a revelation 96-well multi-scanner plate reader (Bio-Rad Laboratories Inc., Munich, Germany). The results obtained were calculated and were represented by survival relative to controls. This experiment was repeated 3 times independently, and statistical analysis was done to obtain the final values.

### 2.7. Cell Invasion and Migration Assay

The cell invasion and migration ability of 2 different colorectal cell lines was evaluated using alginate beads as described previously [52]. For the invasion assay, 1 × 10^6^/mL of each type of cell were cultured in alginate beads in Petri dishes, treated with different concentrations of resveratrol (1, 5, 10 µM) or co-treated with fixed (0.5 µM) Sirt1-ASO or Sirt1-SO, 10 µL/mL Lipofectin transfection reagent and with different concentrations of resveratrol (1, 5, 10 µM) in serum-starved medium for 14 days. After incubation for 14 days, noninvasive cells and alginate beads were removed from the Petri dishes, and migrated cells adhering and forming colonies on the bottom of the Petri dish were fixed with methanol for 10 min, stained with toluidine blue for 5 min, carefully washed two times with PBS and photographed under the light microscope (Zeiss, Jena, Germany). The number of migrated and positively-stained adherent colonies were quantified and evaluated manually by counting all colonies.

### 2.8. Electron Microscopy

The alginate cultures were fixed for 1 h in Karnovsky’s fixative followed by post-fixation in a 1% OsO_4_ solution in phosphate buffer, as previously described [50]. After rinsing and dehydration in ascending alcohol series, the samples were embedded in Epon and ultrathin sections prepared with a Reichert-Jung Ultracut E (Darmstadt, Germany). Ultrathin sections were contrasted with 2% uranyl acetate/lead citrate and examined under a Zeiss transmission electron microscope, Jena, Germany (TEM 10, Institute of Pharmacology, Berlin, Germany), or Jeol 1200 EXII, Akishima Tokyo, Japan (Department of Anatomy and Cell Biology, Martinsried, Germany).

### 2.9. Western Blot Analysis

For Western blot analysis, proteins were extracted from the alginate cultures with lysis buffer (50 mM Tris-HCl, pH 7.2, 150 mM NaCl, 1% (v/v) Triton X-100, 1 mM sodium orthovanadate, 50 mM sodium pyrophosphate, 100 mM sodium fluoride, 0.01% (v/v) aprotinin, 4 µg/mL of pepstatin A, 10 µg/mL of leupeptin, 1 mM phenylmethylsulfonyl fluoride, PMSF) on ice for 30 min, as previously described [54,55]. Total protein concentration was measured with the bicinchonic acid assay system (Uptima, Monlucon, France) using bovine serum albumin as a standard. Samples were further reduced with 2-mercaptoethanol and equal quantities of protein (500 ng/lane), separated under reducing conditions by SDS-PAGE and transferred onto nitrocellulose membranes using a trans-blot apparatus (Bio-Rad). After preincubation in blocking buffer (5% skimmed milk powder in PBS, 0.1% Tween 20) for 2 h, membranes were incubated with primary antibodies at 4 °C overnight, washed three times with blocking buffer and then further incubated with alkaline phosphatase-conjugated secondary antibodies for 1.5 h at ambient temperature. After further washing in 0.1 M Tris, pH 9.5, containing 0.05 M MgCl_2_ and 0.1 M NaCl, specific antigen-antibody complexes were detected using nitro blue tetrazolium and 5-bromo-4-chloro-3-indoylphosphate (*p*-toluidine salt; Pierce).

### 2.10. Isolation of CRC Cell Nuclear Extracts

Cell nuclear extracts were isolated as previously described [56]. Briefly, cells were trypsinized and washed twice in 1 mL of ice-cold PBS. The supernatant was carefully removed. The cell pellet was resuspended in hypotonic lysis buffer containing protease inhibitors and was incubated on ice for 15 min. Then, 12.5 µL of 10% Nonidet P-40 was added, and the cell suspension was vigorously mixed for 15 s. The extracts were centrifuged for 1.5 min. The supernatants (cytoplasmic extracts) were frozen at −70 °C. Twenty five microliters of ice-cold nuclear extraction buffer were added to the pellets and incubated for 30 min with intermittent mixing. Extracts were centrifuged, and the supernatant (nuclear extracts) was transferred to pre-chilled tubes for storage at −70 °C.

### 2.11. Immunofluorescence Microscopy Analysis of Monolayer Cultures

HCT116 and SW480 cells were cultured on glass plates in a monolayer. After fixation with methanol, cells were rinsed with PBS and incubated with bovine serum albumin (BSA) for 30 min. Primary antibodies were diluted 1:50 in PBS/BSA, incubated overnight at 4 °C in a humid chamber, washed three times with PBS/BSA followed by incubation with rhodamine-coupled secondary antibodies for 1.5 h and finally washed again three times with aqua dest. Counter staining was performed with DAPI (49,6-diamidino-2-phenylindole, Sigma) to visualize cell nuclei. Slides were covered with fluoromount mountant and examined under a fluorescent microscope (Leica, Darmstadt, Germany).

### 2.12. Statistical Analysis

Each experiment was performed three times individually with three replicates. Data were expressed as the mean values (±SD) or SEM, as indicated in the figures. Results were analyzed by an unpaired Student’s *t*-test and by one-way ANOVA followed by a *post hoc* test to compare the parameters of each group. Differences were considered to be statistically significant for *p* < 0.05.

## 3. Results

The purpose of this paper was to investigate whether resveratrol/Sirt1-mediated signaling during carcinogenesis plays a role against the growth and metastasis of CRC cells. We used two different well-characterized cell lines, microsatellite instability MSI^+^ (HCT116) and MSI^−^ (SW480) cells, as well as cells that are WT for p53 (HCT116) and mutant (SW480) [57], since 5-Fluorouracil (5FU)-mediated resistance is tightly linked to MSI and p53 status in CRC, derived from colorectal cancer cells, and we evaluated the effects of resveratrol on Sirt1 targeting using Sirt1-ASO on NF-κB signaling pathways in human CRC cells.

### 3.1. Resveratrol Upregulated the Expression of Sirt-1 Protein in CRC Cells in Vitro

**Figure 1:**
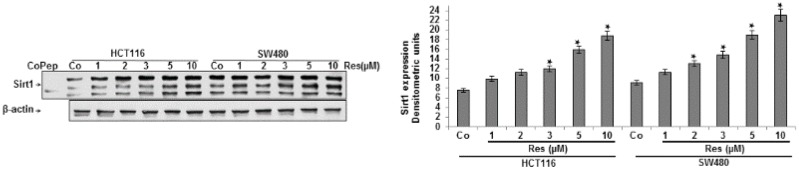
The effects of resveratrol on Sirt1 expression in CRC cells in the alginate tumor microenvironment. Serum-starved HCT116 and SW480 cell lines in alginate culture (1 × 10^6^/mL) were either left untreated or treated with different concentrations of resveratrol alone (1, 2, 3, 5, 10 µM) for 14 days. Whole cell lysates were prepared and analyzed by Western blotting with antibodies against Sirt1. Sirt1 control peptide (Co Pep) was used as a control for Sirt1 antibody specificity. The results shown are representative of three independent experiments. Housekeeping protein β-actin served as a loading control in all experiments. Densitometric evaluation of protein expression as revealed by Western blot analysis was performed in triplicate. Values were compared to the control and statistically-significant values with *p <* 0.05. Significant values are marked with an asterisk (*).

First, we examined whether resveratrol stimulates the expression of Sirt1 in different CRC cells. HCT116 and SW480 cells in alginate culture were either left untreated or treated with different concentrations of resveratrol (1, 2, 3, 5, 10 µM) for 14 days. Whole cell lysates were fractionated and analyzed by immunoblotting using anti-Sirt1 antibodies. As shown in Figure 1, Western blotting and densitometric analysis were performed in triplicate, and treatment with resveratrol alone clearly upregulated the expression of Sirt1 protein in a dose-dependent manner in both CRC cells (HCT116 and SW480).

### 3.2. Inhibitory Effects of Resveratrol on Cellular Proliferation and Viability of CRC Cells Were Abolished by Knockdown of Sirt1

**Figure 2:**
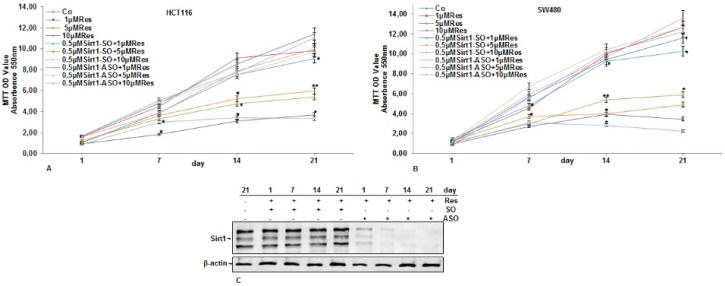
Effects of resveratrol and Sirt1-ASO on the proliferation of CRC cells in alginate culture. Serum-starved HCT116 (**A**) and SW480 (**B**) cell lines in alginate culture (1 × 10^6^/mL) were either left untreated, or treated with different concentrations of resveratrol alone (1, 5, 10 µM), or transfected with 0.5 μM sense oligonucleotide (SO) control, or antisense oligonucleotides (ASO) against Sirt1 in the presence of Lipofectin transfection reagent (10 μL/mL) and co-treated with different concentrations of resveratrol (1, 5, 10 µM) for 21 days in alginate cultures, and cell viability was measured using the MTT method. The results are provided as mean values with standard deviations from at least three independent experiments. Values were compared to the control, and statistically-significant values with *p* < 0.05 are designated by an asterisk (*); *p* < 0.01 designated by two asterisks (**); (**C**) Effects of Sirt1-ASO on Sirt1 expression in CRC cells in alginate culture. Serum-starved HCT116 cells in alginate culture were either left untreated or transfected with 0.5 μM sense oligonucleotide (SO) control or antisense oligonucleotides (ASO) against Sirt1 in the presence of Lipofectin transfection reagent (10 μL/mL) and co-treated with resveratrol (5 µM) for 21 days. Whole cell lysates were prepared and analyzed by Western blotting with antibodies against Sirt1. Housekeeping protein β-actin served as a loading control in all experiments.

HCT116 and SW480 cells in alginate beads (1 × 10^6^/mL) were either left untreated or treated with different concentrations of resveratrol (0, 1, 5, 10 μM), transfected with Sirt1-SO, Sirt1-ASO (0.5 µM) in the presence of Lipofectin (10 µL/mL) or cells were co-treated with different concentrations of resveratrol (1, 5, 10 µM) and with Sirt1-SO, Sirt1-ASO (0.5 µM) in the presence of Lipofectin (10 µL/mL) for 21 days in serum-deprived medium, and cell viability was measured using the MTT assay, as described in the Experimental Section. The tumor cells proliferated continuously in control alginate cultures, whereas resveratrol inhibited the proliferation and increased cell death in HCT116 and SW480 cells in a dose-dependent manner (Figure 2A,B). Treatment of CRC cells with different concentrations of resveratrol and Sirt1-SO (0.5 µM) in the presence of Lipofectin induced growth inhibitory effects on cellular viability similar to resveratrol alone or Sirt1-SO (0.5 µM) alone (not shown). In contrast, knockdown of Sirt1 with ASO abolished the inhibitory effects of resveratrol on cell viability and proliferation similar to CRC cells transfected with Sirt1-ASO (0.5 µM) alone (not shown). Furthermore, to examine, whether knockdown of Sirt1 protein levels with specific ASO are still low 21 days after transfection during tumorigenesis in alginate cultures, we performed Western blot analysis. HCT116 (Figure 2C) and SW480 (not shown) cells in alginate beads (1 × 10^6^/mL) either served as controls or were treated as described above (Figure 2). The collected cells were subjected to immunolabeling with Sirt1 antibody. As demonstrated in Western blot analysis, treatment with resveratrol and Sirt1-SO upregulated the expression of Sirt1 protein in a time-dependent manner (Figure 2C) in both CRC cells. In contrast, treatment with Sirt1-ASO clearly downregulated levels of Sirt1 protein in a time-dependent manner in both CRC cells (Figure 2B), and it keeps 21 days after transfection in alginate cultures (Figure 2C). Taken together, these findings suggest an essential role for Sirt1 in resveratrol-promoting anti-tumorigenic effects in CRC cells.

### 3.3. Resveratrol-Induced Suppression in Migration and Invasion of CRC Cells Is Blocked by Knockdown of Sirt1

**Figure 3:**
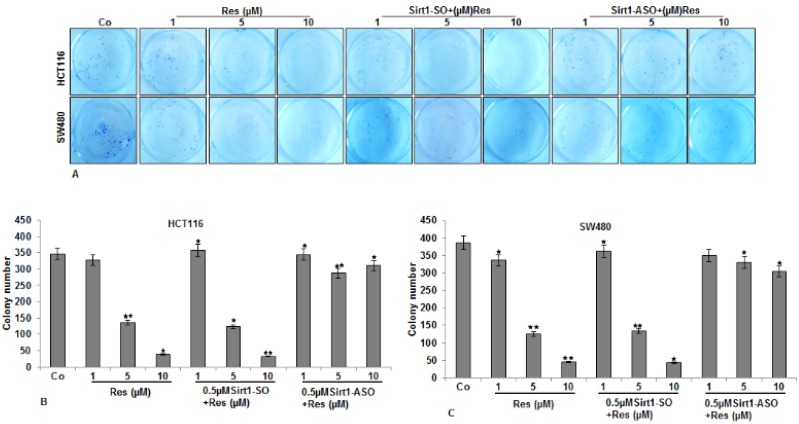
Effects of resveratrol and Sirt1-ASO on the invasion of CRC cells in alginate culture. (**A**) Serum-starved HCT116 and SW480 cell lines in alginate culture (1 × 10^6^/mL) were either left untreated or treated as described above (Figure 2) and emigrated spheroids evaluated by toluidine blue staining after 14 days; (**B**,**C**) The spheroid numbers of emigrated HCT116 (**B**) and SW480 (**C**) cells through alginate beads was quantified after 14 days in culture. The values given are the means ± standard errors of the mean of three replicates. One of three independent experiments is shown. Values were compared to the control, and statistically-significant values with *p* < 0.05 are designated by an asterisk (*); *p* < 0.01 by two asterisks (**).

Next, we investigated whether downregulation of Sirt1 by mRNA modulates the anti-tumor effects of resveratrol against CRC migration and invasion in the 3D alginate-based culture microenvironment, by evaluating through toluidine blue staining. As shown in Figure 3, treatment of the cells with resveratrol alone inhibited migration of HCT116 (Figure 3A,B) and SW480 (Figure 3A,C) cells through the alginate-based matrix in a dose-dependent manner. Interestingly, it was noted that there was no effect of Sirt1-SO on resveratrol-mediated migration inhibition on CRC cells. However, knockdown of Sirt1 with ASO blocked resveratrol-inhibited migration effects and significantly increased the number of migrated cells (Figure 3A–C).

### 3.4. Specific ASO against Sirt1 Blocked Resveratrol-Induced Inhibition of Ki-67 Expression in CRC Cells

**Figure 4:**
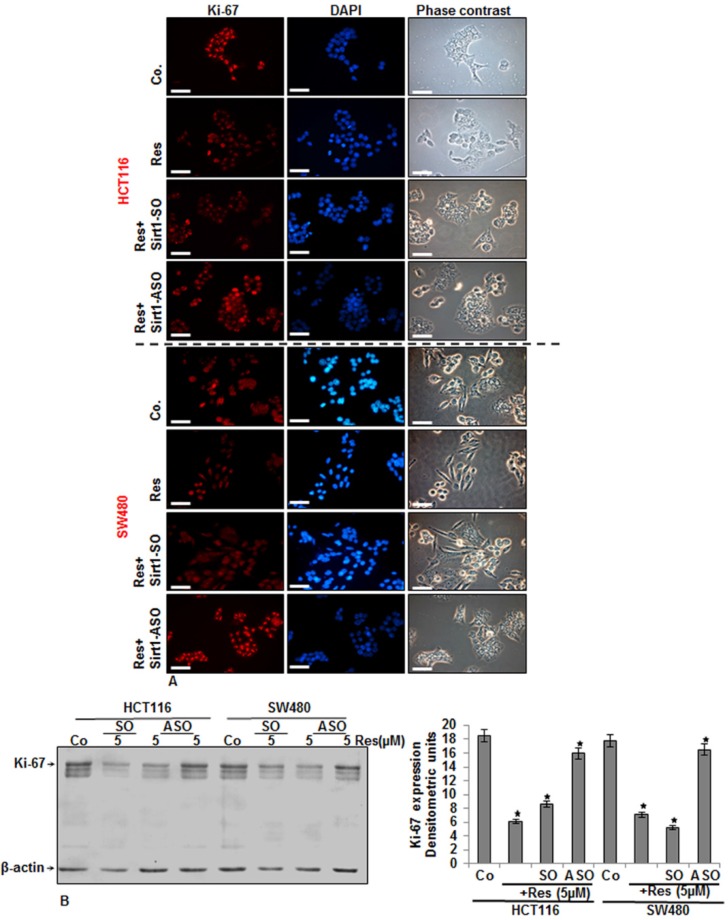
Effects of resveratrol and Sirt1-ASO on Ki-67 in CRC cells in monolayer culture as revealed by immunofluorescence microscopy. Serum-starved HCT116 (**A**) and SW480 (**A**) cell lines in monolayer culture were either left untreated, or treated with resveratrol alone (5 µM), or transfected with 0.5 μM sense oligonucleotide (SO) control, or antisense oligonucleotides (ASO) against Sirt1 in the presence of Lipofectin transfection reagent (10 μL/mL) for 24 h and co-treated with resveratrol (5 µM) for an additional 24 h and fixated with methanol. For immunolabeling, cells were incubated with primary antibodies against Ki-67 followed by incubation with rhodamine-coupled secondary antibodies and counterstaining with DAPI to visualize cell nuclei. Images shown are representative of three different experiments. Magnification 400×; bar = 30 nm; (**B**) Effects of Sirt1-ASO on Ki-67 expression in CRC cells in monolayer culture. Serum-starved HCT116 and SW480 cell lines in monolayer culture were either left untreated or transfected with 0.5 μM sense oligonucleotide (SO) control or antisense oligonucleotides (ASO) against Sirt1 in the presence of Lipofectin transfection reagent (10 μL/mL) and co-treated with resveratrol (5 µM) for 24 h. Whole cell lysates were prepared and analyzed by immunoblotting with antibodies against Ki-67. Housekeeping protein β-actin served as a loading control in all experiments. Densitometric evaluation of protein expression as revealed by Western blot analysis was performed in triplicate. Values were compared to the control and statistically-significant values with *p <* 0.05. Significant values are marked with an asterisk (*).

Ki-67 protein is used as a marker for cell proliferation, and it has been shown that resveratrol inhibits its expression [58]. CRC cells (HCT116 and SW480) either served as controls or were transfected with 0.5 µM Sirt1-SO or Sirt1-ASO in the presence of Lipofectin for 24 h and co-treated with resveratrol (5 µM) for an additional 24 h, and surviving colonies were collected. The results from the immunofluorescence analysis (Figure 4A), as well as the immunoblot assays (Figure 4B) showed that resveratrol alone significantly downregulated the expression of Ki-67 and nuclear localization. Furthermore, transfection with Sirt1-ASO, but not with control Sirt1-SO (Figure 4A,B), inhibited resveratrol-induced suppression of Ki-67 expression in both CRC cell lines, indicating that Sirt1 is one of the main targeting proteins by resveratrol during resveratrol-anti-proliferative effects in CRC cells. Quantitative Western blot analyses by densitometric evaluation performed in triplicate confirmed the immunofluorescence results (Figure 4B). Taken together, these results showed that resveratrol alone significantly downregulated the expression of Ki-67, which is one of the principle mechanisms of inhibition of tumor growth and invasion.

### 3.5. Knockdown of Sirt1 with Specific ASO Suppresses Resveratrol-Upregulated Sirt1 Expression in CRC Cells

**Figure 5:**
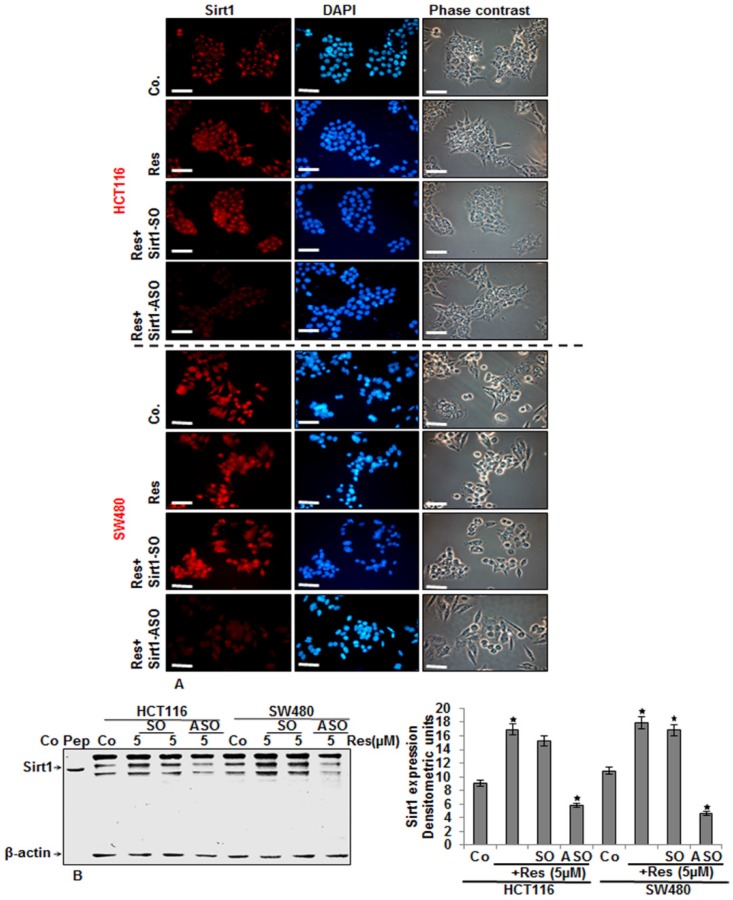
Effects of resveratrol and Sirt1-ASO on Sirt1 expression in CRC cells in monolayer culture as revealed by immunofluorescence microscopy. Serum-starved HCT116 (**A**) and SW480 (**B**) cell lines in monolayer culture were either left untreated or treated as described above (Figure 4). For immunolabeling, cells were incubated with primary antibodies against Sirt1 followed by incubation with rhodamine-coupled secondary antibodies and counterstaining with DAPI to visualize cell nuclei. Images shown are representative of three different experiments. Magnification 400×; bar = 30 nm; (**B**) Effects of Sirt1-ASO on Sirt1 expression in CRC cells in monolayer culture. Serum-starved HCT116 and SW480 cell lines in monolayer culture were either left untreated or treated as described above (Figure 4). Whole cell lysates were prepared and analyzed by Western blotting with antibodies against Sirt1. Housekeeping protein *β*-actin served as a loading control in all experiments. Densitometric evaluation of protein expression as revealed by immunoblot analysis was performed in triplicate. Values were compared to the control and statistically-significant values with *p <* 0.05. Significant values are marked with an asterisk (*).

CRC cell lines (HCT116 and SW480) either served as controls or were treated as described above (Figure 4). The collected cells were subjected to immunolabeling or immunoblotting with Sirt1 antibody. The immunofluorescence analysis (Figure 5A), as well as the immunoblot analysis (Figure 5B) revealed that resveratrol alone or with control Sirt1-SO significantly upregulated the expression of Sirt1 protein and nuclear localization (Figure 5A,B). In contrast, transfection with Sirt1-ASO (Figure 5A,B) significantly inhibited resveratrol-induced upregulation of Sirt1 expression in both CRC cell lines, indicating that Sirt1 is one of the main target proteins by resveratrol during the resveratrol-anti-tumor effect in CRC cells. Quantitative Western blot analyses by densitometric evaluation performed in triplicate confirmed the immunofluorescence results (Figure 5B).

### 3.6. Knockdown of Sirt1 with Specific ASO Blocks Resveratrol-Inhibited NF-κB Activation in CRC Cells in the Alginate Tumor Microenvironment

**Figure 6:**
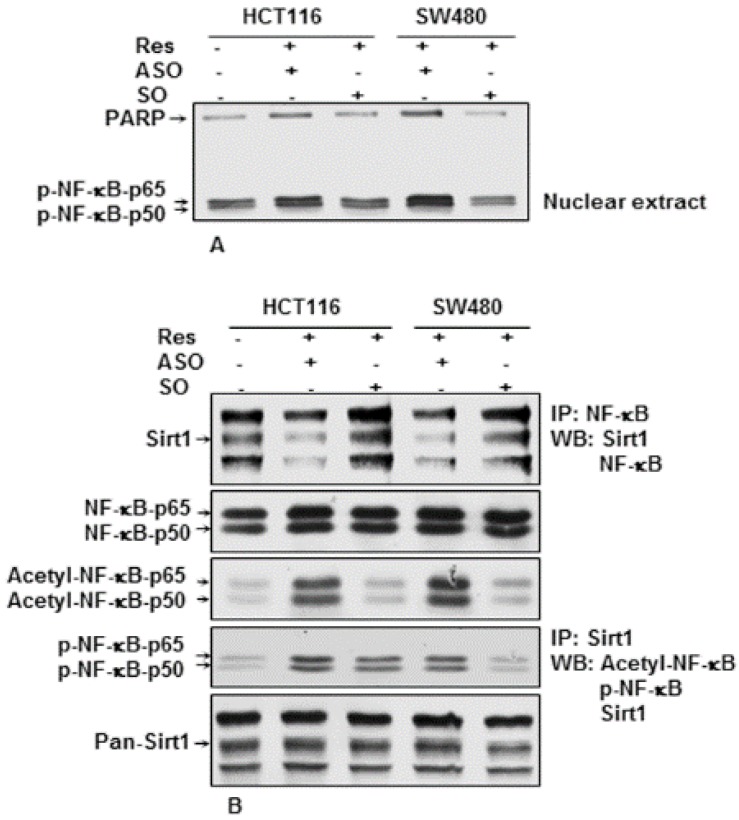
Effects of resveratrol or/and ASO against Sirt1 on NF-κB signaling pathways in CRC cells. (**A**) Antisense oligonucleotide against Sirt1 induced phosphorylation and translocation of NF-κB in nuclear extracts in CRC cells. Serum-starved HCT116 and SW480 cell lines in alginate culture (1 × 10^6^/mL) were either left untreated or transfected with 0.5 μM sense oligonucleotide (SO) control or antisense oligonucleotides (ASO) against Sirt1 in the presence of Lipofectin transfection reagent (10 μL/mL) and co-treated with resveratrol (5 µM) for 14 days. Nuclear cell fractions were prepared and examined by Western blot analysis using antibodies against phospho-NF-κB and nuclear protein poly(ADP-ribose) polymerase as a loading control. Western blots shown are representative of three independent experiments; (**B**) Effects of resveratrol and/or ASO against Sirt1 on acetylation and phosphorylation of NF-κB and Sirt1/NF-κB interaction in CRC cells. After completion of the experiments, whole cell extracts were immunoprecipitated (IP) with anti-NF-κB antibody or anti-Sirt1 antibody and then analyzed by Western blotting (WB) using antibodies against Sirt1, acetyl-lysine and phospho-NF-κB. The same immunoprecipitates were then reblotted with NF-κB or Sirt1 antibody.

To investigate the underlying mechanism of the sensitivity of CRC cells to resveratrol, we examined whether the effects of resveratrol on CRC cells’ growth and metastasis in 3D alginate cultures was associated with the inhibition of NF-κB activation. Several lines of evidence have suggested that NF-κB mediates tumor progression, and the tumor chemoresistance of different tumor cells is known to activate NF-κB expression [59,60]. Our group has shown previously that co-treatment with resveratrol reduced NF-κB activation in IL-1β, but not in ASO-treated normal cells, indicating that Sirt1 suppression on mRNA levels is not reversible by resveratrol and highlighting the essential role of Sirt1 in the resveratrol-specific signaling pathway [61]. CRC cells (HCT116 and SW480) in alginate culture were either left untreated or transfected with 0.5 μM sense oligonucleotide (SO) control or antisense oligonucleotide (ASO) against Sirt1 in the presence of Lipofectin and co-treated with resveratrol (5 µM) for 14 days. Nuclear cell fractions were prepared and analyzed by Western blotting. Transfection with ASO against Sirt1 induced phosphorylation and translocation of NF-κB (p50 and p65) to the nucleus (not shown). Co-treatment with resveratrol suppressed NF-κB activation in Sirt1-SO-treated, but not in Sirt1-ASO-treated cells, indicating that Sirt1 suppression on mRNA levels is not reversible by resveratrol and highlighting the essential role of Sirt1 in inhibiting the NF-κB pathway (Figure 6A).

### 3.7. Downregulation of Sirt1 with ASO Blocks Resveratrol-Induced Sirt1 Association, De-Acetylation and Phosphorylation of NF-κB in CRC Cells

The results of other investigations and our laboratory have shown that stimulation of Sirt1 enzymatic activity by resveratrol correlated with increased de-acetylation of NF-κB, suggesting that both proteins interact together [33,53]. Therefore, next, we performed co-immunoprecipitation assays to examine the interaction of Sirt1 and NF-κB in CRC cells in alginate tumor microenvironment cultures. HCT116 and SW480 cells were either left untreated or treated as described above (Figure 6A). Whole cell extracts were immunoprecipitated with antibody against NF-κB and then immunoblotted with anti-Sirt1 antibodies. As shown in Figure 6B, immunoprecipitates from CRC cells in Sirt1-ASO-/resveratrol-treated cultures revealed strong co-immunoprecipitation of Sirt-1 protein with the NF-κB protein (Figure 6B). In contrast, in untreated controls and Sirt1-SO-/resveratrol-treated cultures, marginal Sirt1 co-immunoprecipitation with the NF-κB protein was observed (Figure 6B). Moreover, the same samples were immunoprecipitated with anti-Sirt1 and probed by Western blotting with antibodies against p-NF-κB, acetyl-lysine and Sirt1. Immunoprecipitates from CRC cells in untreated controls and Sirt1-SO/resveratrol-treated cultures, but not from Sirt1-ASO/resveratrol-treated cultures, revealed inhibition or marginal co-immunoprecipitation of acetylated and phosphorylated NF-κB subunits (p50/p65) (Figure 6B). Taken together, these findings indicate that downregulation of Sirt1 with specific ASO knocked down Sirt1 protein levels and Sirt1-NF-κB complex formation during tumorigenesis in alginate cultures (Figure 6A,B), and this blocked the ability of resveratrol to deacetylate and phosphorylate NF-κB, which may, at least in part, inhibit resveratrol-promoting anti-tumorigenic effects in CRC cells.

### 3.8. Resveratrol-Induced Suppression of NF-κB-Dependent Gene Products Involved in Proliferation and Metastasis is Blocked by Knockdown of Sirt1 in CRC Cells

**Figure 7:**
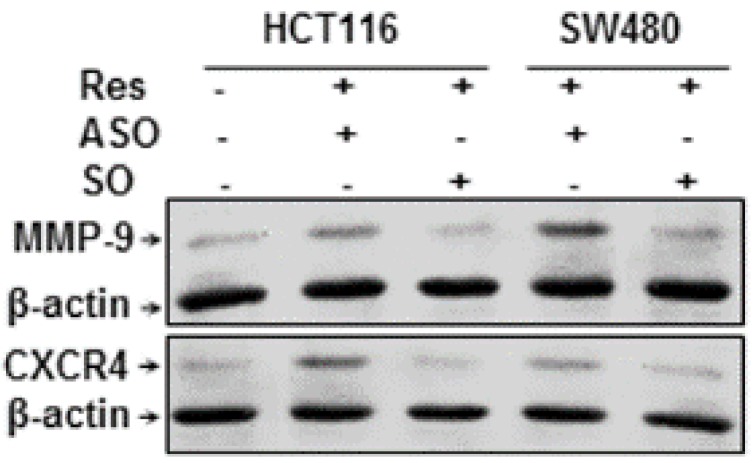
Effects of resveratrol or/and ASO against Sirt1 on NF-κB-regulated gene products involved in proliferation and metastasis in colorectal cancer cells. Serum-starved HCT116 and SW480 cell lines in alginate culture (1 × 10^6^/mL) were either left untreated or treated as described above (Figure 6). Whole cell lysates were fractionated and subjected to Western blotting with antibodies against MMP-9, CXCR4 and β-actin. Housekeeping protein β-actin served as a loading control in all experiments.

It has been reported that NF-κB regulates the expression of genes involved in proliferation, invasion and metastasis [7]. The alginate cultures of CRC cells were either left untreated or treated as described above (Figure 6A). As shown in Figure 7, we examined further the expression of the NF-κB-regulated gene products that are involved in proliferation, invasion (MMP-9) and metastasis (CXCR4). The results of Western blot analysis showed clearly that Sirt1-SO/resveratrol-treated cultures, but not Sirt1-ASO/resveratrol-treated cultures, downregulated the expression of the mentioned proteins in CRC cells. Taken together, these results demonstrate that specific suppression of Sirt1 in CRC cells by Sirt1-ASO, at least in part, is one of the important anti-tumorigenic mechanisms in resveratrol signaling pathways during tumorigenesis.

### 3.9. Resveratrol Induces Epithelial Phenotype (MET) in Colorectal Cancer Cells, but Not in the Presence of ASO against Sirt1 in 3D Alginate Culture

**Figure 8:**
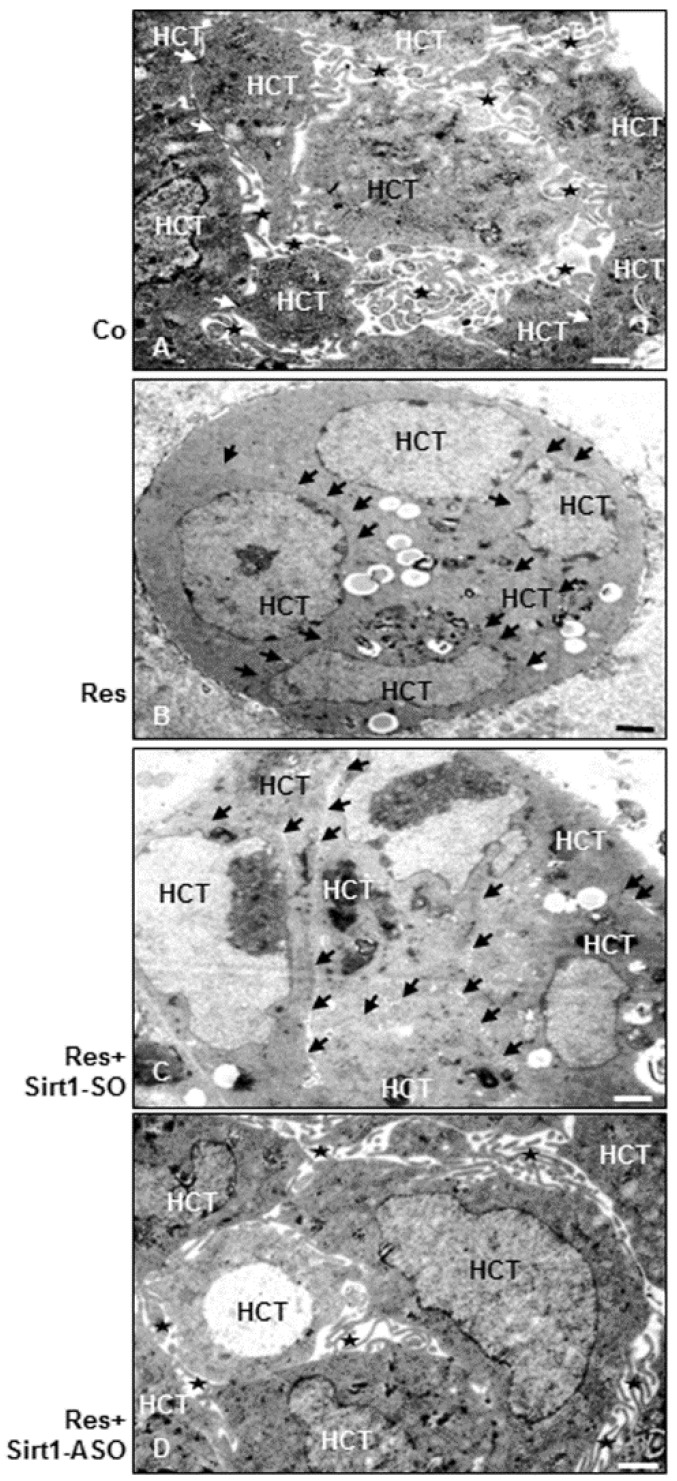
Transmission electron microscopic investigation of cell-phenotype behavior in CRC cells in alginate beads after treatment with resveratrol or/and ASO against Sirt1. Serum-starved HCT116 cells in alginate culture (1 × 10^6^/mL) were either left untreated (**A**), or treated with resveratrol alone (5 µM) (**B**), or transfected with 0.5 μM sense oligonucleotide (SO) control (**C**), or antisense oligonucleotides (ASO) against Sirt1 (**D**) in the presence of Lipofectin transfection reagent (10 μL/mL) and co-treated with resveratrol (5 µM) for 14 days. After 14 days of culture, transmission electron microscopic observations of HCT116 cells in alginate showed in control cultures tumor cells with a planar surface and microvilli (*) on their surface (**A**). Treatment with resveratrol alone or co-treated tumor cells (HCT116) with resveratrol and control-SO showed only a few microvilli, but they had contact planar surface (arrows) (**B**,**C**). Sirt1-ASO- and resveratrol co-treated tumor cells contained abundant microvilli on their surface (**D**). Scale bar = 1 µm.

To examine whether the growth-suppressive effects of resveratrol in CRC colonosphere formation in 3D alginate culture are associated with any ultrastructural cell morphology changes on the cell-cell behavior, transmission electron microscopic evaluations were performed (Figure 8A). After a 14-day culture period, untreated alginate cultures of HCT116, SW480 (not shown) and cells transfected with Sirt1-SO (not shown) showed morphological features of viable cell proliferation and aggregation formation encapsulated in alginate. Cells were mainly round to oval, contained a well-developed rough endoplasmic reticulum, a large Golgi apparatus and other organelles, such as mitochondria, all of which indicate the maintenance of metabolic functions (Figure 8A). CRC cells contained numerous abundant microvilli and cell surface processes (Figure 8A). Opposite to this, in the presence of resveratrol alone or co-treated with resveratrol and transfected with control Sirt1-SO, a conversion to an epithelial morphology was observed. CRC cells were observed to grow in close clusters with almost a planar surface (Figure 8B,C). Furthermore, CRC cells transfected with Sirt1-ASO (not shown) or co-treated with resveratrol exhibited a mesenchymal-like morphology associated with increased abundant membrane extensions, microvilli and long filopodia (Figure 8D). Taken together, these results indicate that the resveratrol/Sirt1 signaling pathway induces an MET (mesenchymal to epithelial transition) phenotype in CRC cells in the alginate tumor microenvironment.

## 4. Discussion

Colorectal cancer is one of the most prevalent malignant tumors in the world, with high rates of recurrence, metastasis and morbidity [1]. Furthermore, it has been reported that tumors are multigenic diseases, and most chemotherapies alone are toxic and ineffective in a large majority of patients and will not have much future. Therefore, new preventive or therapeutic agents that are pharmacologically safe and effective are urgently needed. Resveratrol as a multi-targeted safe natural polyphenolic compound [15] has been linked with chemosensitizing potential and anticancer properties; however, the mechanisms of the complex roles of resveratrol molecular signaling during carcinogenesis are still poorly understood.

The aim of this paper was to determine the mechanisms by which resveratrol suppresses tumor cell growth and metastasis in CRC cells by targeting Sirt1 protein and regulating the NF-κB signaling pathway. It has been shown that resveratrol exerts its positive effects by promoting the protein deacetylase enzyme silent information regulator 2/Sirtuin 1 (Sirt1) activity [62,63] through an allosteric mechanism [64] and has emerged as an agent that can extend the life span, probably through the delay of most chronic illnesses, including cancer [65], by preventing genome instability [66,67].

We showed that resveratrol inhibited the proliferation, invasion and metastasis of CRC cells (HCT116 and SW480) in 3D-alginate cultures, and this was blocked by knockdown of Sirt1 by using ASO, suggesting that Sirt1 suppression on mRNA levels is not reversible by resveratrol, highlighting the crucial role of this enzyme. Furthermore, it has been recently shown that resveratrol suppresses the proliferation and growth of gastric cancer cells in a Sirt1-dependent manner *in vitro* and *in vivo* [68]; however, we have shown in this report for the first time the inhibition of CRC cell proliferation through the resveratrol/Sirt1 pathway in 3D alginate cultures. Our findings are consistent with reports that show resveratrol can block the proliferation of diverse cancer cells through modulation of cell cycle regulatory gene products, induction of apoptosis by upregulation of p53 and inhibition of anti-apoptotic gene products [20,58,68,69].

Recent reports from our and other laboratories have revealed that alginate culture provides an excellent tumor microenvironment, and CRC cells encapsulated in 3D alginate cultures proliferated with detachment of the tumor cells from the alginate and formed metastases, suggesting that this model is suitable to investigate the initial steps of tumorigenesis, as it is comfortable to perform *in vitro* the sequential steps of CRC metastasis mimicking the microenvironment of CRC tumor *in vivo* [52,70]. We further noticed that the proliferation marker Ki-67 and tumor-promoting factors (MMP9, CXCR4) were significantly downregulated by resveratrol in CRC cells. These results are consistent with previous studies, which showed that resveratrol inhibits angiogenesis, proliferation and metastasis in different kinds of tumors [71,72,73].

To examine the mechanism of resveratrol signaling, we first investigated whether the NF-κB transcription factor pathway was involved. We found that downregulation of NF-κB activation and nuclear translocation by resveratrol, which is constitutively activated in CRC cells, could be a possible mechanism. Furthermore, we could also show that NF-κB-regulated gene products (CXCR4 and MMP-9), which are expressed by CRC cells, were also significantly downregulated by resveratrol. Interestingly, we have further demonstrated that inhibition of NF-κB nuclear translocation and NF-κB-regulated gene products by resveratrol was reversed through Sirt1 suppression on mRNA levels, thus suggesting that more than one mechanism may be involved in the anti-tumor and anti-proliferative activity of resveratrol. Indeed, several lines of evidence suggest that resveratrol has various chemoregulatory effects in different kinds of cells and signaling pathways in a Sirt1-dependent mode [68,74,75], as demonstrated by knockdown experiments. We also showed that the negative effect of resveratrol on CRC cells was, at least in part, regulated by the inhibition of transcription factor NF-κB acetylation and phosphorylation. Moreover, immunoprecipitation and Western blotting results clearly showed functional and physical interactions between NF-κB and Sirt1, highlighting that this interaction may play an important role in regulating resveratrol’s anti-tumor effects in CRC. Furthermore, the inhibitory effect of resveratrol on acetylation and phosphorylation of NF-κB was abolished through knockdown of Sirt1 by mRNA.

It has been shown that resveratrol is one of the most potent Sirt1 activators by provoking a structural conformational change in Sirt-1, inducing the acetylated substrate and NAD, consequently resulting in an increased enzymatic activity [33], which has also been associated with apoptosis [76]. Indeed, many Sirt1 substrates are transcription factors and key regulators known to participate in embryonic growth and in neoplasia (*i.e.*, p53, NF-κB, Ku70 and FoxOs) [77]. In our earlier studies, we also demonstrated that resveratrol is a potent suppressor of the NF-κB signaling pathway in different cell types [78,79,80], which has been closely linked to inflammation and cancer [7] and was found to promote proliferation and metastasis [15,81]. The physical interaction between NF-κB and Sirt1 indicates that NF-κB could mediate resveratrol/Sirt1-dependent anti-proliferative and anti-metastasis function in CRC cells, and this was consistent with the finding by knockdown of Sirt1 by using ASO. Our findings are further in agreement with studies that resveratrol downregulates proliferation, invasion and metastasis in glioma, lung carcinoma and breast tumors [71,72,73,82]. Our findings indicate clearly that Sirt1 serves as a tumor suppressor in CRC cells in alginate culture, and the toxic effect of resveratrol on the tumor cells appears to be, at least in part, the result of specific resveratrol-caused activities of a cardinal favorable effect of Sirt1 protein activation.

It has been shown that the anti-aging effects of resveratrol might be mediated via different mechanisms, like anti-oxidation, anti-cyclooxygenase activity and anti-free radical activity, effects on cell cycles *in vitro* and *in vivo* and through activation of Sirt1, which is a conserved determinant of life span upon calorie restriction [33,77,83,84]. In contrast, resveratrol has been found to suppress growth and proliferation in diverse tumor cells by various molecular targeting [85,86]. Indeed, several lines of evidence have reported that Sirt1 may have controversial complex roles, either as a cancer suppressor or as a cancer promoter [37,38,41]. It has been considered that phytopharmaceuticals can modulate different cell-signaling pathways by the regulation of different molecular targets in diverse cell types. The reciprocal chemomodulatory effects of resveratrol in various cell types may be, at least partially, due to the bifurcated roles of Sirt1 in tumors (promoting and/or suppressing tumorigenesis), suggesting an issue of the nature of genes that are integrated in the genome maintenance of cells. It has been suggested that Sirt1 plays an important role in epigenetic modifications of chromatin structure and DNA repair by deacetylation [87,88]; thus, Sirt1 controls the cellular response to stress. The condition and activity of Sirt1 protein in tumor cells may therefore probably play a role to cause the cell response to epigenetic stress and treatment.

Recently, we have shown that resveratrol, at least in part, suppresses the proliferation and metastasis of CRC cells by upregulation of intercellular junctions in alginate cultures [51]. The ultrastructural electron microscopic examinations in this study have shown clearly that CRC cells’ surface revealed in the control contained numerous abundant microvilli and cell surface processes and showed numerous intercellular junctions on the planar surface. Interestingly, through treatment with resveratrol or co-treatment with control SO and resveratrol, a dramatic conversion to an epithelial ultrastructural morphology with numerous intercellular junctions on the planar surface was observed. In contrast, in the presence of Sirt1-ASO or co-treatment with resveratrol and Sirt1-ASO, the CRC cells underwent ultrastructural changes with highly metastatic signs, e.g., the development of microvilli on nearly the entire surface of the cells and microfilaments, indicating that the inhibition effects of resveratrol on CRC cell migration and proliferation are dependent on Sirt1. According to previous studies, tight cell-cell contact between tumor cells is associated with more cell integrity and tumor metastasis suppression [89,90]. These findings are consistent with other studies, which have shown that cancer cell surface morphological changes are closely involved with malignant behavior and proliferation of tumors [90,91]. Indeed, it seems that the intercellular adhesion junction may have a regulatory effect on cell aggregation and stabilization of the tumor cells, leading to malignity and metastasis originating from cancer cells [90,92,93].

Evidence from *in vivo* studies has shown that resveratrol is absorbed after oral administration and metabolized and conjugated to yield resveratrol glucuronide [94]; its peak concentration was achieved after 60 min [95] and revealed the good tissue distribution and clearance of resveratrol [58]. Furthermore, it has been reported that a combination therapy of traditional chemotherapy agents with natural compounds has the potential to target more signaling pathways involved in tumorigenesis and limits the toxicity effects from chemotherapeutic agents, and several studies have shown the benefit of this combination therapy in colorectal cancer cells [96,97].

## 5. Conclusions

In conclusion, we found that resveratrol, by targeting the subcellular Sirt1 signaling pathway, is one of the main mechanisms of the anti-tumor effects of resveratrol on CRC cells. The bifurcated role of the resveratrol/Sirt1 signaling pathway with various signaling targets in tumor cellular processes suggests the therapeutic potential of resveratrol in the prevention/treatment of human CRC.
